# Squibbian Inconsistency

**Published:** 1887-11

**Authors:** 


"SQUIBBIAN INCONSISTENCY."

The profession is indebted to the *American Lancet* for the above suggestion. It occurs in an editorial in the November number signed "R. W. P.," and is used to express the writer's idea of Dr. Squibbs' endeavor to convince the profession that the fluid extract of rhamnus frangula, made by Squibbs, is better than an extract of rhamnus pursiana made by others; notwithstanding the dose of the former is larger, and the extract is sold for more than the latter, both having the same effect, as claimed by Dr. S. The writer scores Dr. Squib unmercifully, accusing him of having used the profession to his own mercenary ends, gulling them, and finally insulting them, when he finds that he can do so successfully, no longer. The article certainly shows up "the veteran Squib" (vide a certain Presidential address) in an ugly light. But as it is not our funeral, and as we had our say on the subject some time ago—which "say" is quoted in the *Lancet*—we have nothing further to add just now. We commend the article in the *Lancet* to our readers as *breezy* and pungent reading—sharp enough to set one's teeth on edge. Whew! but it's red hot!

